# Health-seeking behaviour of male foreign migrant workers living in a dormitory in Singapore

**DOI:** 10.1186/1472-6963-14-300

**Published:** 2014-07-10

**Authors:** Weixian Lee, Andy Neo, Sandra Tan, Alex R Cook, Mee Lian Wong, Joshua Tan, Andrew Sayampanathan, Daniel Lim, Shin Yong Tang, Wei Leong Goh, Mark I-Cheng Chen, Chwin Ho

**Affiliations:** 1 Yong Loo Lin School of Medicine, National University of Singapore, Singapore, Singapore; 2 Saw Swee Hock School of Public Health, National University of Singapore, MD3, 16 Medical Drive, Singapore 117597, Singapore; 3 HealthServe Community Clinic, Singapore, Singapore; 4 Department of Clinical Epidemiology, Institute of Infectious Disease and Epidemiology, Tan Tock Seng Hospital, Singapore, Singapore

**Keywords:** Migrant worker, Health survey, Health seeking behaviour, Singapore

## Abstract

**Background:**

Foreign workers’ migrant status may hinder their utilisation of health services. This study describes the health-seeking behaviour and beliefs of a group of male migrant workers in Singapore and the barriers limiting their access to primary healthcare.

**Methods:**

A cross-sectional study of 525 male migrant workers, ≥21 years old and of Indian, Bangladeshi or Myanmar nationality, was conducted at a dormitory via self-administered questionnaires covering demographics, prevalence of medical conditions and health-seeking behaviours through hypothetical scenarios and personal experience.

**Results:**

71% (95%CI: 67 to 75%) of participants did not have or were not aware if they had healthcare insurance. 53% (95%CI: 48 to 57%) reported ever having had an illness episode while in Singapore, of whom 87% (95%CI: 82 to 91%) saw a doctor. The number of rest days was significantly associated with higher probability of having consulted a doctor for their last illness episode (p = 0.026), and higher basic monthly salary was associated with seeing a doctor within 3 days of illness (p = 0.002). Of those who saw a doctor, 84% (95%CI: 79 to 89%) responded that they did so because they felt medical care would help them to work better. While 55% (95%CI: 36 to 73%) said they did not see a doctor because the illness was not serious, those with lower salaries were significantly more likely to cite inadequate finances (55% of those earning < S$500/month). In hypothetical injury or illness scenarios, most responded that they would see the doctor, but a sizeable proportion (15% 95%CI: 12 to 18%) said they would continue to work even in a work-related injury scenario that caused severe pain and functional impairment. Those with lower salaries were significantly more likely to believe they would have to pay for their own healthcare or be uncertain about who would pay.

**Conclusions:**

The majority of foreign workers in this study sought healthcare when they fell ill. However, knowledge about health-related insurance was poor and a sizeable minority, in particular those earning < S$500 per month, may face significant issues in accessing care.

## Background

International movement of migrant labour is an increasing global phenomenon, with approximately half of about 175 million migrants around the world being migrant workers [1]. In Southeast Asia, Singapore is a major receiving country for migrant labour. As of December 2010, there were an estimated 1,113,200 migrant workers (35% of Singapore’s workforce) in Singapore. Of these, 685,400 were semi-skilled or unskilled workers, including 293,400 in construction [2] and 131,000 in the shipyard sector, [3] and come largely from South East Asia (Malaysia, Thailand, Indonesia, the Philippines and Myanmar), South Asia (India and Bangladesh) and China [4,5].

The increasing number of migrant workers in high risk occupations in Singapore, [6] and their long working hours may elevate the risk of occupational accidents [7]. They are also at risk from occupational skin and lung diseases, and work-related musculoskeletal disorders. Additionally, outbreaks of infectious diseases such as typhus, dengue and pneumonia have been documented, possibly due to the high-density living conditions and occasionally less than satisfactory sanitary living conditions [8-10]. The high risk of health related problems may be compounded by issues with adequate and timely access to healthcare, particular in lower income workers. While Singapore has a highly accessible primary care network, migrant workers in Singapore were no longer eligible for government subsidized medical care since 2007, [11] and fees at private sector primary care clinics range from S$18 to S$50 per consult (S$1 = 0.8 US dollars as of May 2014), which may be costly to some migrant workers.

Singapore’s Employment of Foreign Manpower Act (Chapter 91A) does mandate employers to procure medical insurance for the foreign employee’s medical expenses, with coverage of at least $15,000 per 12-month period of the foreign employee’s employment [12]. The Work Injury Compensation Act (WICA) provides injured employees with a means to claim compensation for injuries sustained during work; the worker only has to prove that he suffered an accident or incurred any diseases due to work for the employer [13]. However, there have been media reports of instances where medical treatment and sick leave have not been commensurate with the severity of workplace accidents, [14] and anecdotal accounts of workers not wanting to report sick due to fear of losing a day’s pay for missing work or even of being laid off if they take too many sick days [15]. However, it is unclear how widespread such issues may be, whether migrant workers are sufficiently aware of their rights to medical care and compensation, and how this might or might not affect their health-seeking behaviour.

Other research has documented utilization patterns of migrant workers at the emergency department, [16-18] but there is a dearth of information on their health-seeking behaviour, and what factors may predispose to inadequate or delayed access to healthcare in Singapore. In collaboration with a non-governmental organisation (NGO), HealthServe, which has outreach activities in a large dormitory for migrant workers and an offsite clinic nearby, we conducted a cross-sectional survey to ascertain health-seeking behaviour, describe reasons for and possible barriers to seeking care, as well as identify any potential vulnerable groups within this population of migrant workers.

## Methods

### Research site and study population

The research was conducted at an all-male dormitory for migrant workers in Jurong, Singapore, near a major industrial area. The dormitory housed approximately 7000 migrant workers from shipyard and construction industries under various companies, and had residents of several nationalities with a significant representation in the migrant work force in Singapore, including mainly Indians (45%) and Bangladeshis (35%), and a significant number of Myanmars (10%).

### Selection of participants and data collection

We first observed the dormitory on 2 weekday evenings to establish patterns of human traffic, and identify key sites and timeframes to conduct the survey. The gantry point where all the residents pass through when entering and leaving the dormitory was identified as the ideal location for recruitment of survey participants. We observed that, from 5 pm to 10 pm, 5334 out of the estimated 7000 residents (76%) returned from work, with the peak flow (2257 (32%)) occurring from 7 to 8 pm. About 60% returned between 7 pm to 10 pm, which were the opening hours of the function room made available to us by our NGO partner HealthServe for conducting our survey.

We then conducted the survey over 3 weekday evenings. We systematically approached one in ten men as they were about to enter the dormitory gantry to invite them to participate, with inclusion criteria being: male; 21 years old and above; Non-Singaporean and non-permanent resident; having a valid work permit for 3 months or more; being of Indian, Bangladeshi and Myanmar ethnicity; and being able to understand English, Bangladeshi, Myanmar, Hindi, Tamil, or Telugu. Verbal consent was obtained and those who agreed undertook the survey in a quiet room. Small tokens of appreciation (biscuits and drinks) were handed out to those who completed the questionnaire. No identification was taken, but participants who reported that they had already taken part once were excluded to prevent repeat participation.

### Questionnaire design, translation and administration

Insights from a qualitative research study involving in-depth interviews with 16 migrant workers from the same dormitory [19] were used to aid the design of a questionnaire comprising three major sections. The first covered personal and socio-demographical information like age, nationality, marital status, education, salary and working conditions. Another section covered previous health-seeking experience based on participants’ response to a previous illness or injury, and their reasons for and against seeking medical attention. A final section covered how they would respond to four hypothetical illness or injury scenarios: (A) an upper respiratory tract infection (URTI), (B) an URTI lasting three days and complicated by high fever (38°C), (C) a worksite injury to the foot with some pain but no functional impairment, and (D) the same worksite injury but with pain so severe as to cause functional impairment. The self-administered questionnaire was translated into Bangladeshi, Myanmar, English, Hindi, Tamil, and Telugu, reflecting the linguistic backgrounds of our study population, with accuracy of translation verified through back-translation to English. Facilitators underwent half a day of training on questionnaire administration, and were assisted by volunteer translators from amongst the workers at the survey site to answer participants’ queries.

### Sample size calculation and statistical analysis

We calculated our target sample size of 500 to give confidence intervals within ±5% of an estimate on the proportion who seek healthcare, which was assumed to lie between 30% to 90% for a given scenario or condition. Chi-squared tests were used when comparing differences in health-seeking behaviour by various socio-demographic factors, with a p-value of less than 0.05 indicating a statistically significant result. We also evaluated predictors of inadequate or delayed access to healthcare for two outcomes of interest – seeing a doctor, and seeing a doctor within 3 days during their most recent illness episode. Odd ratios (ORs) from multivariate logistic regression incorporating all variables significant at a level of p < 0.10 on univariate analysis are also presented. Analyses were performed using Stata for Windows, version 11 (Stata Corporation, College Station, Texas, USA).

### Ethics approval

The study was approved on 22/1/2013 by the ethics review board of the National University of Singapore (IRB reference 12-503).

## Results

**Figure 1:**
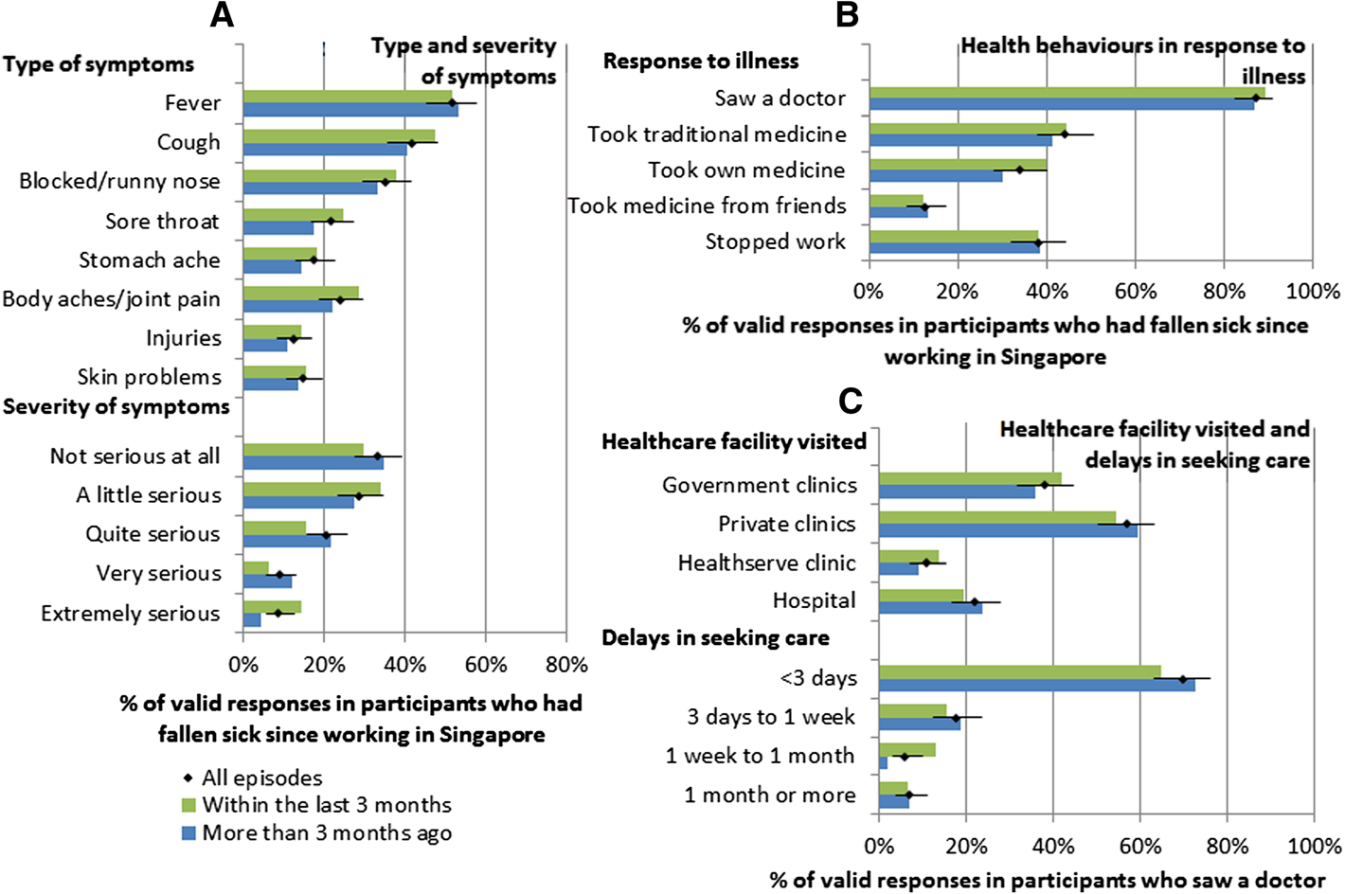
**Characteristics of and response to most recent illness episodes while working in Singapore, with stratification by timing of illness episode. A)** type and severity of symptoms and **B)** response to illness in participants who had fallen sick since working in Singapore; **C)** healthcare facility visited and delays in seeking care in participants who saw a doctor. Diamonds are proportions for all episodes (where error bars denote 95% confidence intervals), and green and blue bars are for episodes within the last 3 months and more than 3 months ago respectively.

Of 1,305 persons approached, we identified 540 (41%) eligible participants who agreed to participate. After excluding 15 incomplete questionnaires, data from 525 participants was available for analysis (Table 1). The majority were of Indian (49%) or Bangladeshi (45%) nationalities, and in the shipyard industry (79%). Most were young (63% aged 20 to 29, and only 5% were 40 or older) and single (64%), but 77% were financially supporting 4 or more people in their home country. Most (89%) participants had a basic salary of S$999 or less per month; 74% worked more 45 hours or more a week and 48% reported 2 or less rest days per month. Only 29% knew they had an existing healthcare insurance plan for their work in Singapore, while 40% and 31% respectively reported that they did not have or did not know if they had one.273 participants (53%) reported having fallen sick while working in Singapore. The most recent illness episode (Figure 1A) commonly involved fever (53%) and respiratory symptoms like cough (43%), blocked/runny nose (35%), and sore throat (20%); about a quarter reported body aches and joint pains, and 12% also reported injuries. The high prevalence of these conditions support their subsequent use in constructing our hypothetical illness and injury scenarios. The majority of episodes were mild, with 62% describing the symptoms as either not serious or only a little serious. The vast majority (87%) did see a doctor for their illness (Figure 1B), but only 38% stopped work while ill. A substantial proportion used traditional medicines (44%) and their own medicines (34% bought over-the-counter or retained from previous prescriptions and 12% from their friends). In those who saw a doctor (Figure 1C), private general practitioners and workplace doctors (57%) were the most visited, followed by government clinics (38%) and hospitals (22%), and a substantial proportion (11%) had visited the HealthServe clinic. While 70% consulted a doctor within 3 days, the remainder delayed seeking care for 3 or more days. We found no significant differences in the type of symptoms, response to illness and healthcare facilities visited, and also no significant trend in the severity of symptoms or in the delays in seeking care in the stratified analysis by timing of illness episodes.

**Table 1:** Socio-demographics and healthcare insurance characteristics of participants (N = 525)

**Characteristic (No. of valid responses)**		**n**	**%**
Language used in survey **(N = 525)**	Bengali	228	43
	Tamil	150	29
	English	69	13
	Telugu	40	8
	Myanmar	29	5
	Hindi	9	2
Age in years **(N = 525)**	21 to 29	333	63
	30 to 39	168	32
	40 and above	24	5
Nationality **(N = 525)**	Indian	256	49
	Bangladeshi	237	45
	Myanmar	32	6
Marital status **(N = 521)**	Single	331	64
	Married	184	35
	Widowed/Separated	6	1
No. of people supported **(N = 505)**	3 or less	116	23
	4 to 6	270	53
	7 or more	119	24
Industry **(N = 518)**	Shipyard/Marine	409	79
	Construction	73	14
	Others	36	7
Highest education **(N = 523)**	Primary or less	54	10
	Secondary	249	48
	Post-secondary	220	42
Basic monthly salary **(N = 520)**	S$499 or less	195	37
	$500 to $999	269	52
	$1,000 or more	56	11
Average working hrs/wk **(N = 516)**	Less than 45 hours	131	26
	45 to 65 hours	265	51
	More than 65 hours	120	23
No. of rest days/mth **(N = 517)**	2 days or less	247	48
	3 to 4 days	163	31
	5 or more days	107	21
Duration in Singapore **(N = 516)**	2 years or less	199	39
	3 to 4 years	89	17
	5 or more years	228	44
Healthcare insurance plan **(N = 525)**	Yes	153	29
	No	209	40
	Do not know	163	31

**Figure 2:**
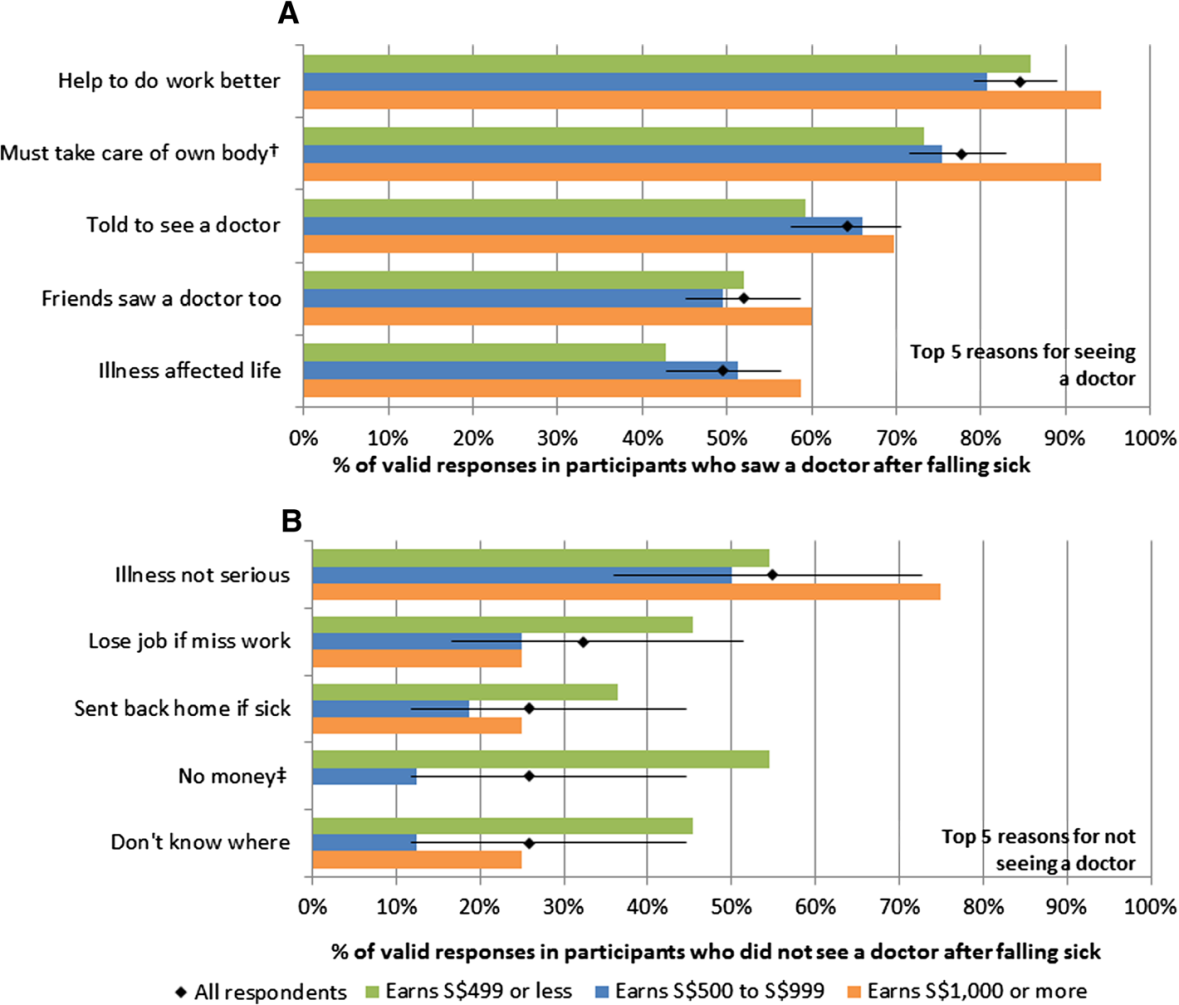
**Top 5 reasons for seeing a doctor (A) and for not seeing a doctor (B), with stratification by basic monthly salary.** Diamonds give the proportion of all valid responses, with 95% confidence intervals as error bars, while coloured bars give the corresponding proportions for the three income groups: those earning S$499 or less (green), S$500 to S$999 (blue) and $S1,000 or more (orange) per month. Symbols † and ‡ are reasons where the frequency is different between the income groups at p < 0.05 and p < 0.01 respectively by chi-squared test for trend.

Table 2 shows that those with more rest days were significantly more likely to see a doctor when sick (p = 0.026, chi-squared test for trend); participants were also more likely to seek care if fever was one of the symptoms (p = 0.066). On multivariate analysis, the number of rest days remained significantly associated with seeing a doctor (OR 1.8, 95%CI: 1.1–3.1, p = 0.020), while the association with fever was borderline significant (OR 2.1, 95%CI: 1.0–4.6, p = 0.054). There was a significant trend where those supporting more people at home (p = 0.017) and those with lower salaries (p = 0.002) were less likely see a doctor within 3 days as compared to all others who fell sick, whereas fever (p < 0.001) and cough (p = 0.045) were associated with seeing a doctor within 3 days. On multivariate analysis, the basic monthly salary (OR 1.6, 95%CI: 1.1–2.4, p = 0.021) and fever (OR 2.7, 95%CI: 1.5–5.0, p = 0.001) remained significantly associated with seeing a doctor but not cough (OR 0.9, 95%CI: 0.5–1.6, p = 0.628), while the association with number of people supported was borderline significant (OR 0.7, 95%CI: 0.5–1.0, p = 0.072).Figure 2A shows the top 5 reasons cited for seeing a doctor, with 85% of participants (95%CI: 79 to 89%) agreeing with the statement that they did so because they felt medical care would help them to work better, and another 78% (95%CI: 72%–83%) that they "must take care of their own body" (i.e. take responsibility for their own health). Of 31 participants who did not see a doctor when ill, 55% (95%CI: 36 to 73%) responded that they did not do so because they did not deem their illness to be serious. However, 32% (95%CI: 17 to 51%) had concerns about being sent home if sick. In addition, we found important differences when stratifying the analysis by income groups. Those with higher income levels were significantly more likely to agree that they saw a doctor because they "must take care of their own body" (p = 0.032, chi-squared test for trend). In those who did not see a doctor, those with lower income were far more likely to cite financial concerns (p < 0.001), with 55% of those earning S$499 or less per month agreeing that they did not see a doctor because they had "no money".

**Table 2:** Association between selected factors and seeking medical care, and seeking medical care within 3 days based on valid responses to most recent illness episode while working in Singapore

**Factor**		**No. who fell sick**	No. who saw a doctor (%*)	**p-value**	No. who saw a doctor within 3 days (%*)	**p-value**
**Age in years**	21 to 29	177	156 (88)	0.258†	95 (54)	0.845†
	30 to 39	84	70 (83)		43 (51)	
	40 and above	10	10 (100)		6 (60)	
**Nationality**	Indian	137	119 (87)	0.538†	78 (57)	0.391†
	Bangladeshi	108	96 (89)		52 (48)	
	Myanmar	26	21 (81)		14 (54)	
**Marital status**	Single	171	148 (87)	0.729†	91 (53)	0.668†
	Married	94	82 (87)		49 (52)	
	Widowed/Separated	4	4 (100)		3 (75)	
**No. of people supported**	3 or less	60	51 (85)	0.783‡	37 (62)	0.017‡
	4 to 6	139	124 (89)		77 (55)	
	7 or more	60	52 (87)		24 (40)	
**Industry**	Shipyard/Marine	215	187 (87)	0.976†	109 (51)	0.544†
	Construction	35	30 (86)		20 (57)	
	Others	16	14 (88)		10 (63)	
**Highest education**	Primary or less	29	27 (93)	0.606‡	17 (59)	0.469‡
	Secondary	124	107 (86)		67 (54)	
	Post-secondary	117	102 (87)		60 (51)	
**Basic monthly salary**	S$499 or less	90	78 (87)	0.899‡	33 (37)	0.002‡
	$500 to $999	138	120 (87)		84 (61)	
	$1,000 or more	40	35 (88)		24 (60)	
**Average working hrs/wk**	Less than 45 hours	67	57 (85)	0.654‡	32 (48)	0.292‡
	45 to 65 hours	137	120 (88)		74 (54)	
	More than 65 hours	65	57 (88)		37 (57)	
**No. of rest days/mth**	2 days or less	121	101 (83)	0.026‡	63 (52)	0.393‡
	3 to 4 days	91	78 (86)		45 (49)	
	5 or more days	56	54 (96)		34 (61)	
**Duration in Singapore**	2 years or less	89	74 (83)	0.277‡	45 (51)	0.208‡
	3 to 4 years	48	43 (90)		21 (44)	
	5 or more years	130	115 (88)		76 (58)	
**Healthcare insurance plan**	Yes	81	73 (90)	0.538†	45 (56)	0.847†
	No	111	94 (85)		57 (51)	
	Do not know	79	69 (87)		37 (47)	
**Type of symptoms**	Fever	135	123 (91)	0.066†	88 (65)	<0.001†
	Cough	109	99 (91)	0.165†	66 (61)	0.045†
	Blocked/runny nose	93	78 (84)	0.252†	49 (53)	0.969†
	Sore throat	57	48 (84)	0.475†	34 (60)	0.233†
	Stomach ache	45	39 (87)	0.920†	24 (53)	0.876†
	Body aches/joint pains	62	54 (87)	0.923†	28 (45)	0.145†
	Injuries	32	27 (84)	0.595†	18 (56)	0.667†
	Skin problems	39	34 (87)	0.963†	20 (51)	0.851†
**Severity of symptoms**	Not serious at all	88	78 (89)	0.192‡	50 (57)	0.434‡
	A little serious	75	69 (92)		40 (53)	
	Quite serious	53	41 (77)		23 (43)	
	Very serious	24	21 (88)		13 (54)	
	Extremely serious	23	19 (83)		12 (52)	

*As % of all who had illness episode while working in Singapore; p-values by †chi-squared test and ‡chi-squared test for trend.

**Figure 3:**
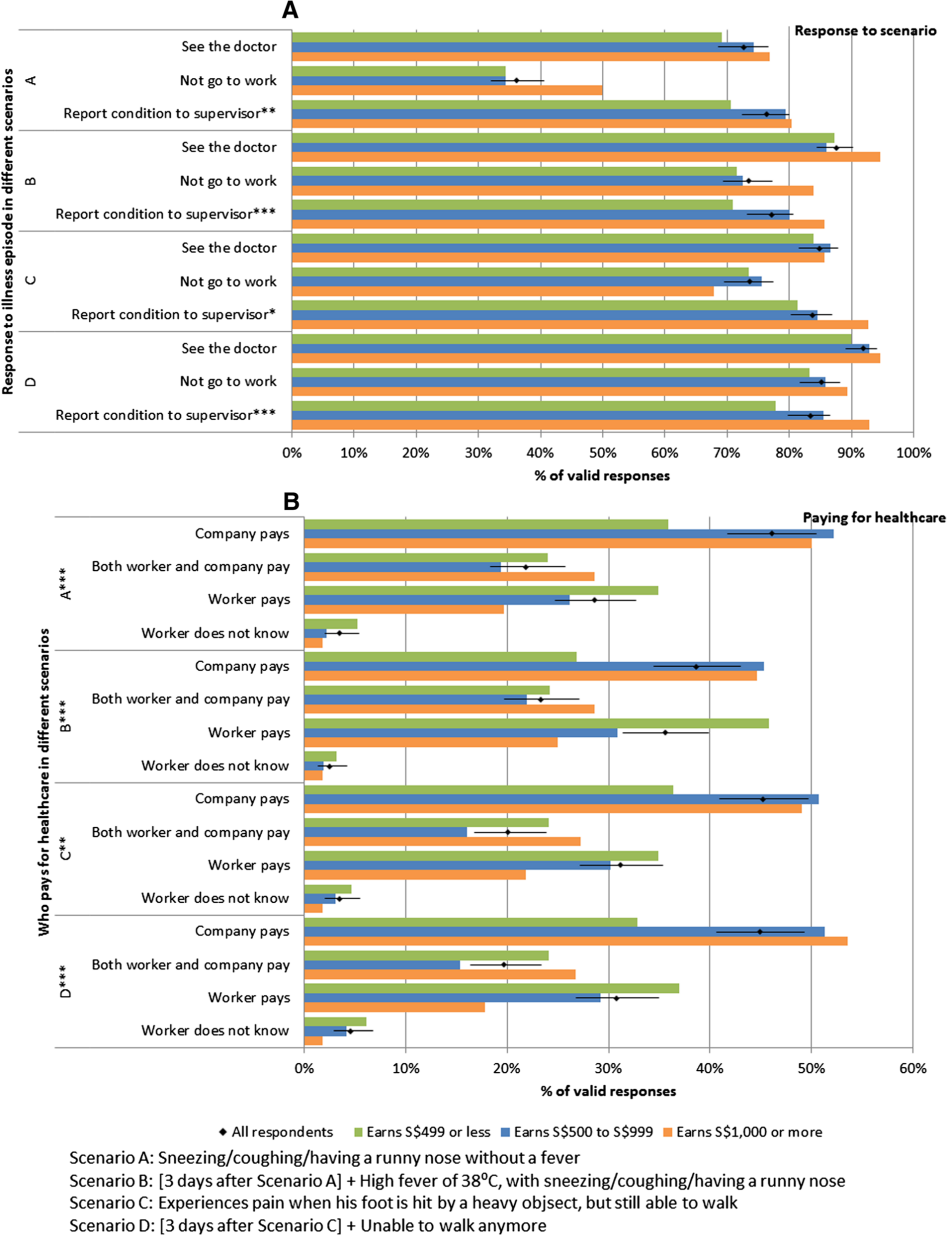
**Responses to illness episode (A) and paying for healthcare (B) in four hypothetical scenarios, with stratification by basic monthly salary.** Diamonds give the proportion of all valid responses, with 95% confidence intervals as error bars, while coloured bars give the corresponding proportions for the three income groups: those earning S$499 or less (green), S$500 to S$999 (blue) and $S1,000 or more (orange) per month. Symbols *, ** and *** are differences with p < 0.10, p < 0.05 and p < 0.01 by chi-squared test for trend **(A)** and chi-squared test **(B)**.

Figure 3 shows the responses to various scenarios, with A and C being situations which may or may not require professional help, and B and D being serious developments arising from A and C respectively where medical attention would clearly be warranted; in addition, Scenarios C and D were clearly work-related whereas Scenarios A and B were not. When comparing the more serious to less serious scenarios (Figure 3A), more participants would not go to work (73% versus 36% in B versus A; 85% versus 74% in D versus C) and more participants would seek care (88% versus 73% in B versus A; 92% versus 85% in D versus C) in both non-work and work related scenarios respectively. However, 42 participants (8%) reported that they would not see a doctor even if they hurt their foot so badly they could not walk, 75 (15%) would continue to work, and 85 (17%) would not notify their supervisors of their condition. Likewise, on having a high fever after being ill for 3 days, 64 (12%) would not see a doctor, 135 (27%) would continue to work, and 118 (23%) would not notify their supervisors. On stratifying by monthly salary, we found that those with higher income were more likely to see a doctor, not go to work, and report to their supervisor. However, in line with our previous analysis (Table 2), the differences in the proportions that would see a doctor were not statistically significant by income groups. There was however, a statistically significant trend for those with higher income to notify their supervisor in scenarios A, B and D. Moreover, Figure 3B shows that income was significantly associated with the worker’s understanding of who would pay for their healthcare, with workers who earned less more likely to believe that they would have to pay on their own, or be uncertain about who would pay. Overall, between 39% and 46% said their company would pay for their medical expenses, but across all four scenarios, more than half believed they would either have to co-pay or pay on their own.

## Discussion

While there have been hospital-based studies describing patterns of health-services utilisation by migrant workers [16,18], this is the first attempt to document health-seeking behaviour in this often neglected population outside of the healthcare setting in Singapore. While utilisation of health services by this migrant population was relatively high, we noted potentially vulnerable groups which may either delay or avoid accessing medical care altogether. Lower income workers, in particular, also had financial concerns about affordability of care, and had the least confidence that their company would be responsible for their medical expenses.

In our study population, the proportion which saw a doctor was consistently high for all conditions and symptoms including injuries, musculoskeletal symptoms, skin problems, febrile illness and respiratory symptoms. The consistently high access to care may have been supported by an onsite private sector clinic within the dormitory compound and a volunteer-run clinic by our NGO partner which was within 15 minutes walking distance. In contrast, a study in Songkhla province, Thailand, found that the proportion who sought care varied widely by type of symptoms, with health-seeking for respiratory symptoms being particularly low (less than 3% of the workers surveyed) [20]. In that study, the perception that symptoms were not serious was cited as a major reason for not seeking medical care [20]. Incidentally, our study also found "illness not serious" as the most important factor for not seeing a doctor (Figure 2B), although there was no association between severity of symptoms and seeking medical care. The overall proportion which saw a doctor for their most recent illness (87%) also compares favourably with a similar study in Beijing where only 36% did so. However, it must be noted that the study in Beijing referred to illnesses in East Asian migrants over a two week period [21], and the health-seeking behaviour of that population and the predominantly South Asian population of our study may be very different. Authors from that study suggest their findings could be attributed to low levels of healthcare insurance and the healthcare policy in China, where migrants from rural communities are classified as temporary residents while in the city, leading to difficulties in accessing care. In our case, although less than a third reported knowing that they had a healthcare insurance plan, 38 to 46% expect their company to pay across the various scenarios in Figure 3. While substantially better than the situation described in Beijing where 94% of the workers denied having healthcare insurance, Singapore’s Employment of Foreign Manpower Act (Chapter 91A) actually mandates that all such expenses be borne by the employer for all foreign workers [12]. As such, there is clearly a gap between the law and either what is practiced or the worker’s knowledge of their entitlement.

Like the other two studies, [20,21] we identified some potentially vulnerable groups. The study from Thailand demonstrated how undocumented workers were far less likely to seek care. Our study, which only included documented workers, found that the only independent predictor of not seeing a doctor was having less rest days per month. However, we did find that lower income was significantly associated with delayed access to care (i.e. not seeing a doctor within 3 days, or not at all); notably, lower monthly per capita household income was also identified as a risk factor for poorer access in the study from Beijing [21]. Furthermore, we showed that in those who did not see a doctor, lower income participants were much more likely to cite financial concerns, and also that such participants were much less likely to notify their supervisors of their condition, and more likely to believe they were responsible for their own healthcare costs in our hypothetical illness/injury scenarios. Moreover, a substantial minority would not notify their supervisor of a serious work-related injury (17%), and not see a doctor despite running a high fever with prolonged respiratory symptoms (12%). Therefore, while financial concerns may not be a barrier to eventual access to healthcare in most of those surveyed, it may be affecting timely access, which would exacerbate conditions like pneumonia and make them harder to treat. In addition, the reluctance to notify supervisors, particularly in lower income workers, may reflect possible communication issues between supervisors and workers that could compromise work-site safety.In terms of policy implications, the most commonly cited reasons for seeking care were the need to "take care of themselves" and to "help them do their work better" (Figure 2A). We believe this suggests most migrant workers would be motivated to seek care when required, as well as adopt other practices important for their health and safety. What is therefore important is to sufficiently educate them on the issues identified, including their entitlement to medical care paid for by their employers, and situations which warrant timely medical attention. The Ministry of Manpower has in recent years included an educational programme highlighting to incoming migrant workers some of their rights alongside key messages on work safety, and what could be done would be to emphasize some of the above points, as well as focus on the vulnerable groups, in particular lower income workers. In addition, these programmes may have to be supplemented by working with large private sector healthcare chains which service migrant workers, peer educators, and access to a course for redress in the event they are treated unfairly, such as designated hotlines for counselling and advocacy services like those provided through our NGO partner HealthServe, or through government channels by the Ministry of Manpower, with provisions made for those only conversant in their native languages.

Our work has several limitations. The survey was restricted to only one dormitory which housed only documented male migrant workers due to the logistical difficulties of accessing migrant workers without prior outreach work, which in this case had been done by our NGO partner. Workers in this dormitory were primarily of three nationalities, and predominantly from the shipyard industry, which would affect the generalisability of our findings. Prior work by our NGO partner may also have made this group more knowledgeable or even improved health seeking behaviour in these migrant workers. Notably, the Ministry of Manpower lists about 30 such migrant worker dormitories around Singapore, and there are also migrant workers not housed in dormitories, and undocumented workers which are difficult to access due to their illegal status. A more detailed study involving a wider range of living quarters, nationalities and industries is hence needed. We also acknowledge that our study suffered from possible biases from a high non-response rate. Better response rates would require alternative recruitment strategies, such as going room-to-room to perform surveys or working through employers. Finally, we were unable to assess migrant workers’ perceptions of healthcare services given the wide diversity of private, government and NGO options which might be accessed by these workers.

## Conclusion

In conclusion, while the majority of the workers we surveyed were able to access care, we identified possible delays in access in a vulnerable group of lower income workers, inadequate knowledge about healthcare insurance plans, and the presence of a sizeable minority who would not seek care when presented with potentially serious health problems. While more representative studies are needed, these are important insights amenable to improvements through appropriate agencies working to provide better education and access to information among migrant workers.

## Competing interests

The authors declare that there is no conflict of interest or, alternatively, disclosing any conflict of interest that may exist.

## Authors’ contributions

LWX participated in the design of the study, performed field work, analysed data and drafted the manuscript. AN participated in the design of the study, performed field work, and helped to draft parts of the manuscript. ST helped to analyse data, edit and revise the manuscript. ARC helped to edit and contribute inputs to the manuscript. WML supervised field work and contributed to the study design and survey instrument. JT and DL contributed to the study design, survey instrument, and parts of the manuscript. TSY and GWL contributed to the study design, manuscript and supervised field work. MC contributed to the study design, data analysis, drafted the manuscript, and supervised field work. HC contributed to the study design, coordinated and performed field work, analysed data and wrote parts of the manuscript. All authors read and approved the final manuscript.

## Pre-publication history

The pre-publication history for this paper can be accessed here:

http://www.biomedcentral.com/1472-6963/14/300/prepub
